# Congenital Pulmonary Airway Malformation in an Adult Male: A Case Report with Literature Review

**DOI:** 10.1155/2015/743452

**Published:** 2015-07-08

**Authors:** Dipti Baral, Bindu Adhikari, Daniel Zaccarini, Raj Man Dongol, Birendra Sah

**Affiliations:** ^1^SUNY Upstate Medical University, East Adams Street, Syracuse, NY 13210, USA; ^2^College of Medical Sciences, Bharatpur 44207, Nepal

## Abstract

Congenital pulmonary airway malformation (CPAM) is a rare cystic lung lesion formed as a result of anomalous development of airways in fetal life. Majority of the cases are recognized in neonates and infants with respiratory distress with very few presenting later in adult life. A 24-year-old male with history of three separate episodes of pneumonia in the last 6 months presented with left sided pleuritic chest pain for 4 days. He was tachycardic and tachypneic at presentation. White blood count was 14 × 10^9^/L. Chest X-ray showed left lower lobe opacity. CT angiogram of thorax showed a well-defined area of low attenuation in the left lower lobe with dedicated pulmonary arterial and venous drainage and resolving infection, suggesting CPAM. He underwent left lower lobe lobectomy. Histopathology confirmed type 2 CPAM. CPAM is a rare congenital anatomic abnormality that can present with recurrent infections in adults. As a number of cases remain asymptomatic and symptomatic cases are often missed, prevalence of CPAM might be higher than currently reported.

## 1. Background

Congenital pulmonary airway malformation (CPAM), previously known as congenital cystic adenomatoid malformation (CCAM), is a developmental lesion of the lung comprising single or multiple cysts of uniform or varying sizes arising from anomalous growth of airways. Most of the cases are identified in infants and neonates with respiratory distress. CPAM can be a cause of pulmonary hypoplasia, severe nonimmune fetal hydrops, and fetal death [1]. On rare occasions, CPAM can present in adulthood with recurrent chest infections, pneumothorax, hemoptysis, or dyspnea [2]. CPAM has been found to be associated malignancies. Ignorance about the existence of this lung condition can lead to missed and delayed diagnosis. We report a rare case of a 24-year-old male who was diagnosed with CPAM during the work-up of recurrent pneumonia.

## 2. Case Summary

A 24-year-old male presented to the hospital with four-day history of moderate left sided chest pain radiating to the back. The chest pain got worse with deep inspiration. He denied fever, chills, cough, hemoptysis, night sweats, weight loss, and recent travel. Past medical history was significant for three episodes of left lower lobe pneumonia in the past 6 months. He was treated initially with ceftibuten and azithromycin and then with a course of oral levofloxacin and most recently with amoxicillin-clavulanic acid for recurring symptoms of cough, pleuritic chest pain, and subjective fever. Currently, he was taking meloxicam as needed for chest pain. Past surgical history included right inguinal hernia repair five years ago. There was no family history of cancer, early death, or cardiac disease. He had immigrated from Guatemala four years ago and was single and unemployed. He denied any high-risk sexual behaviors or drug abuse in the present or past. He drank two beers about once or twice per week and denied smoking history. His differential diagnoses at this point include lung abscess, tuberculosis infection, foreign body aspiration, HIV with opportunistic infection, congenital immunodeficiency states, and congenital developmental anomaly of the lung.

On examination, he was tachycardic with a pulse rate of 101/min and was tachypneic at 22/min. Rest of the physical examination including respiratory examination was normal. Labs revealed complete blood counts of 14 × 10^9^/L with 75% neutrophils. Basic metabolic panel and liver function tests were normal. Urine legionella antigen was negative, as well as antibodies to human immunodeficiency virus. His chest X-ray showed left lower lobe opacity. He was started on ceftriaxone and azithromycin for community acquired pneumonia and was admitted to the floor. Tuberculin skin test was positive with 18 mm induration at 72 hours. Interferon gamma release assay was negative. Blood cultures demonstrated no growth for 5 days.

**Figure 1:**
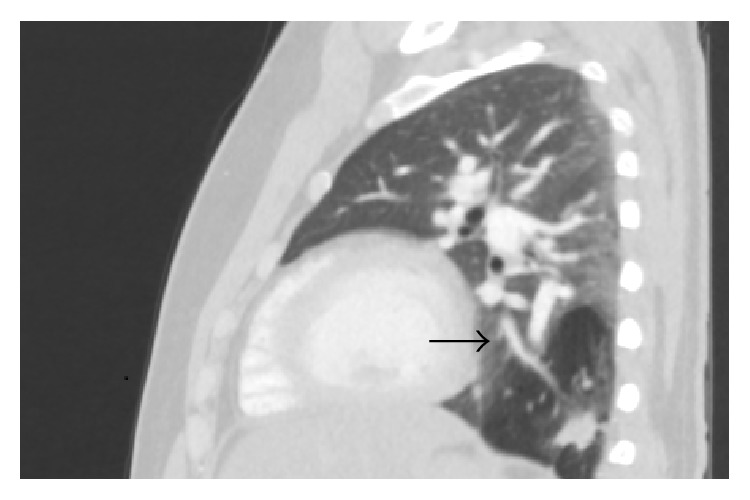
CT thorax sagittal image showing hypodense lesion in the left lower lobe posteriorly with resolving infiltrates within. Arrow: pulmonary vein branch.

**Figure 2:**
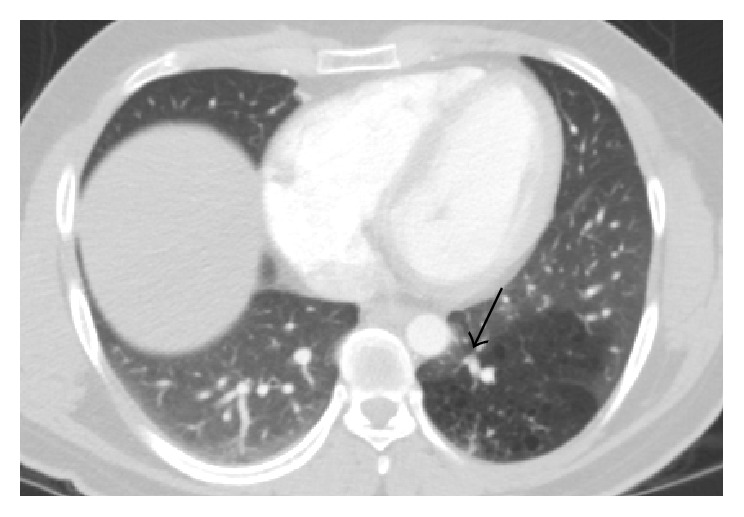
CT angiography shows dedicated pulmonary artery and vein supplying the hypolucent area. Small cysts can be appreciated within the hypolucent area.

**Figure 3:**
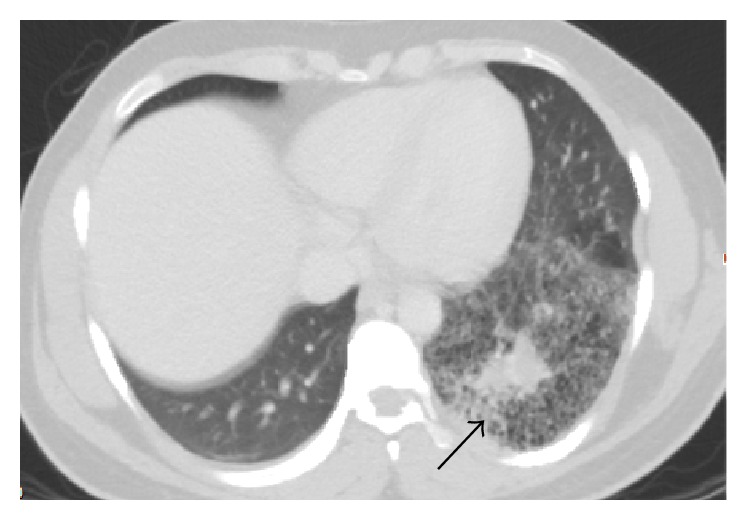
CT scan 4 months ago showing infiltrates in the left lower lung.

CT angiogram of thorax showed 9 cm well-defined area of low attenuation in the left lower lobe (Figure 1) with infiltrates inside. This lesion demonstrated a dedicated pulmonary artery and pulmonary vein (Figure 2); these vessels were emerging from the hilar region. No systemic arteries or anomalous arterial supply was identified within the lesion. There was no pleural involvement or abnormal lymphadenopathy. A radiologic diagnosis of congenital pulmonary airway malformation (CPAM) was made. Review of previous chest X-rays and computed tomography (CT) of the thorax from the time of his previous episodes of pneumonia revealed various degrees of consolidation in left lower base in this particular area (Figure 3). CT abdomen pelvis did not show any abnormal intra-abdominal masses or pathology but showed some hepatic steatosis.

**Figure 4:**
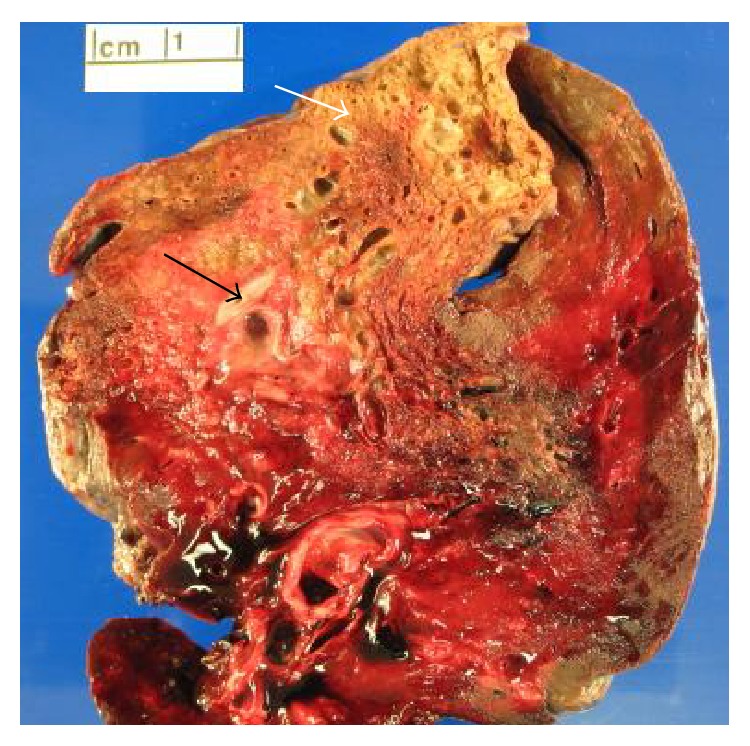
Gross photograph showing multiple air filled microcysts at periphery of lung (white arrow) and a larger cyst (black arrow).

**Figure 5:**
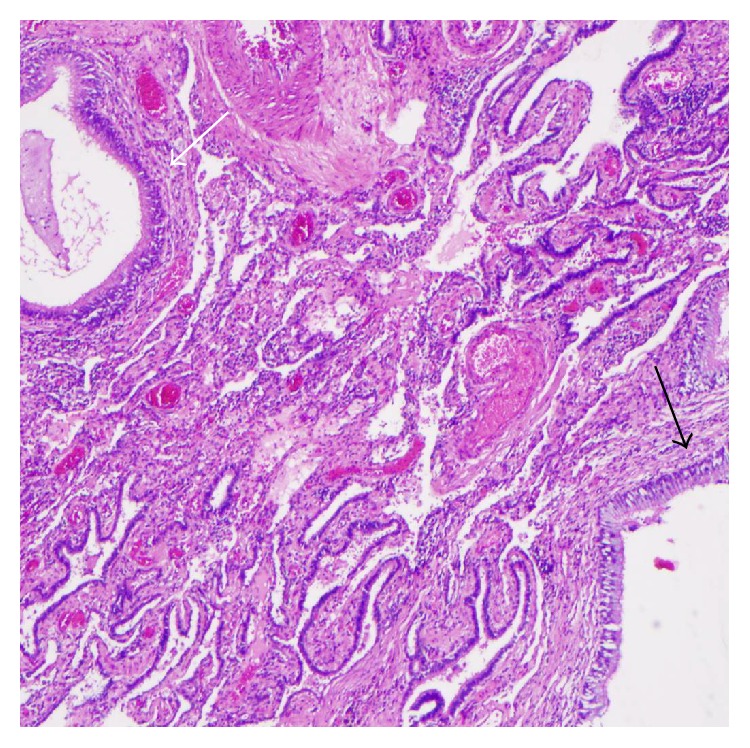
Higher power view of largest cyst (black arrow) showing columnar ciliated epithelium and adjacent smaller cyst (white arrow) with similar lining.

**Figure 6:**
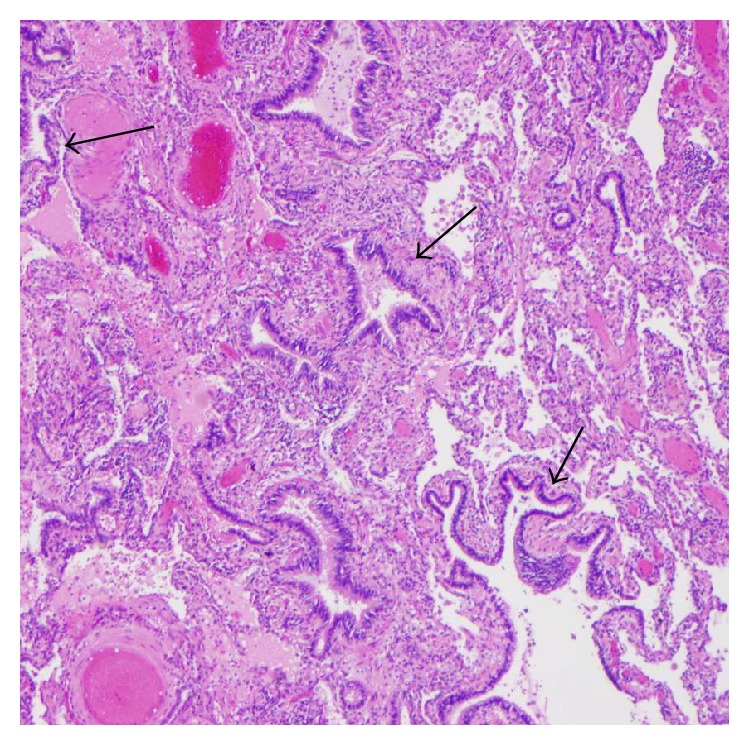
Numerous bronchiole-like structures (black arrows).

As his CT images were highly suggestive of congenital cystic lung lesion, surgical excision was planned to prevent further episodes of pneumonias. Bronchoscopy prior to the surgery revealed normal segmental airways in the left lower lobe. Initially left thoracoscopy was tried; however posterolateral thoracotomy was required for better visualization of the involved anatomy. Upon direct visualization, the complete lobe was involved in chronic inflammation. The lesion had no abnormal arterial supply from aorta or below the diaphragm and was connected through air passages. Left lower lobectomy was done. On gross examination, a relatively well-demarcated lesion with a 1.5 × 1.5 cm thin walled cyst with inspissated mucus within and multiple air filled microcysts at the peripheral aspect of the cyst was noted (Figure 4). Microscopic examination revealed larger cyst with columnar ciliated epithelium (Figure 5) and dispersed bronchiole-like structures within the alveolated parenchyma (Figure 6). The pathologic diagnosis was consistent with type 2 CPAM. There was no major surgical complication. He developed a small hydro pneumothorax postoperatively, which resolved on its own. Patient has been doing well 12 months after the diagnosis.

## 3. Discussion

Congenital pulmonary airway malformation involves increased proliferation and cystic dilatation of different parts of the airways. CPAM comprises around 25% of all congenital lung lesions [3] with an estimated incidence of 1 in 25,000–35,000 pregnancies [4]. CCAM was first described in 1949 by Chin and Tank [5]. Stocker et al. [6] initially classified CCAM into three types in 1977 based on the size and number of the cysts. In 1994, Stocker further expanded the CCAM classification into five categories (Table 1). There are five stages of fetal lung development-embryonic phase, pseudoglandular phase, canalicular phase, saccular phase, and alveolar phase. Types 0–3 originate during the pseudoglandular stage of lung development and type 4 originates during the saccular stage of lung development. This expanded reclassification of CCAM, now renamed CPAM, demonstrates that, as you progress from type 0 to type 4, the main pathologic origin moves from the bronchus, to bronchiole, and then to alveolar tissue. Accordingly, the epithelium varies from pseudostratified to cuboidal to low-cuboidal and simple squamous [7]. It is important to note that frequently there is an overlap between the different types [8]. Type 1 and type 4 CPAM, which have larger cysts, are difficult to differentiate from cystic pleuropulmonary blastoma because of its cystic nature [9].

Histologically the different types of CCAM can usually be distinguished. Type 1 is the commonest type comprising 50–65% of all cases and is characterized by single or multiple larger cysts more than 2 or 3 cm in diameter lined by pseudostratified ciliated columnar epithelium and, sometimes, mucinous type epithelium [2, 7, 10, 11]. Type 2 lesions are characterized by multiple, uniform small (<2–2.5 cm) terminal bronchiole-like cysts lined by cuboidal to columnar epithelium [10]. Our case demonstrated these features and was diagnosed as type 2 CPAM. Type 2 is frequently associated with other congenital lesions [8]. Type 3 CPAM consists of bronchiole-like structures lined by ciliated cuboidal epithelium separated by alveolus-sized structures. Cysts in type 3 CPAM are small and not grossly visible [8]. This type usually involves an entire lung and has spongy appearance with bulk gland-like structures [6].

The exact mechanism of the formation of CPAM is still unknown. No clear hereditary association has been derived so far. However, it has been related to chromosomal abnormalities like trisomy 18 and hereditary renal dysplasia [8]. Some authors believe these lesions develop during the sixth and seventh week of fetal development from arrested growth of localized portions of bronchial tree while others have a concept that they are hamartomatous growth of the bronchial tree [12, 13]. Mutation disrupting TTF-1 (thyroid transcription factor-1), a factor expressed in bronchial and alveolar epithelium that regulates lung epithelial differentiation, has also been considered for the development of CPAMs [2]. Abnormal airways in CPAM have been shown to express high levels of HoxB5 (Homeobox protein) compared to normal lung tissues. Normally HoxB5 gene encodes a protein that regulates normal lung development by working as a sequence-specific transcription factor. This is a part of the developmental regulatory system that provides cells with specific positional identities on the anterior-posterior axis. So it has been postulated that this abnormal expression of HoxB5 gene could also be responsible for the development of CPAM by causing aberrant airway branching patterns [3].

For the most part, CPAM presents with acute respiratory distress in neonates and infants, but occasionally it can remain unnoticed until adolescence or later life [14]. McDonough et al. identified 42 cases of CPAM in the literature up until February 2012 presenting at an age greater than 17 years with equal prevalence in males and females [2]. We found five more cases of CPAM recognized in that age group in the English literature review from February 2012 to February 2015 [15–19]. Almost 44% of CPAM patients are found to have lower lobe lung lesions, primarily unilateral [3]. The most common clinical presentation in adults is recurrent pulmonary infection, pneumothorax, hemoptysis, fever, and dyspnea [2]. Our patient presented with recurrent pneumonia and persistent chest pain in the same location of the lung. 24% of all 42 CPAM cases identified by McDonough were asymptomatic with only radiologic abnormalities. Morelli et al. in their literature review found 9 asymptomatic cases among 45 cases (20%); their review included CPAM cases between ages 6 months and 65 years [20]. Hence, it is difficult to estimate the prevalence of CPAM in general adult population. Other congenital lesions like bronchopulmonary sequestrations, lobar emphysema, renal dysgenesis, intestinal atresia, esophageal cysts, congenital cardiac disease, and so forth have been associated with CPAM [21] and can cause various presenting symptoms depending on the involved organ system. Among the 5 types, type 2 has been found to frequently coexist with these congenital lesions [20].

CPAM is diagnosed by CT scans or MRI of chest. CPAM can be diagnosed prenatally by ultrasonography and is categorized into two groups based on the size of the cysts. Echogenic and solid cysts with diameter <5 mm are microcystic lesions and those with one or more cysts with diameter >5 mm are macrocystic lesions [22, 23]. However ultrasonography can misdiagnose other pathologies like congenital diaphragmatic hernia, bronchopulmonary sequestration, lung atresia, tracheal atresia, and bronchial stenosis as CPAM. Therefore MRI should be the diagnostic imaging choice during prenatal life [24, 25]. Incidence of prenatal diagnosis has increased with the increased use of ultrasound; however it is important to remember that up to 56% of these lesions regress later [8]. The recommended diagnostic test for CPAM in postnatal life is CT scan of the chest [9, 24, 26, 27]. CPAM appears as a large cyst or a cluster of cysts filled with gas or liquid resembling a solid mass in CT scans [28]. CT scan findings vary depending upon the type of CPAM and clinical presentation.

The differential diagnosis of CPAM in adults includes pulmonary sequestration, bronchogenic cysts, and acquired cystic lesions. Bronchogenic cysts arise as an abnormal budding from the primitive tracheobronchial tube. One-fourth of bronchogenic cysts are intrapulmonary, while the rest occur in the mediastinum. Intrapulmonary cysts are usually located in the lower lobes [1]. They are usually unilocular and contain bronchial cartilage, smooth muscle, and mucous gland histologically. Bronchogenic cysts usually do not communicate with alveoli, while adenomatoid malformations do [11]. Pulmonary sequestration is seen as a mass of pulmonary tissue that does not connect with the bronchial passages and has an anomalous blood supply [10]. If the mass is outside the pleura it is defined as extralobar pulmonary sequestration (ELPS). It is called intralobar if it shares pleura with the lung. ELPS is usually detected in the prenatal and neonatal period while late childhood and adulthood diagnosis is common with ILPS [1]. Pulmonary sequestrations can be ruled out radiologically as they have anomalous systemic arterial supply arising from thoracic or abdominal aorta unlike CPAM. It is also wise to be aware that acquired cystic lesions can occur in Ehlers-Danlos syndrome and should be included in the differential diagnosis [29].

It is estimated that approximately 1% of CPAMs, particularly types I and IV, transform into malignancy although the exact incidence is unknown [30]. Mucous cells in type 1 CPAM have tendency to undergo malignant changes [31]. The most common malignancy associated in adults is bronchioloalveolar carcinoma; however, other malignancies like rhabdomyosarcoma, pleuropulmonary blastoma, and adenocarcinoma of lung have been recognized as well [32]. Malignant transformation might start during uterine life with the transformation of epithelial cells in CPAM tissues to atypical epithelial cells via the EGFR pathway. The atypical cells can then progress to papillary predominant adenocarcinoma. The detection of these atypical epithelial cells in the pathological examination of resected tissue emphasizes the importance of complete surgical resection [16].

Due to the risk of malignant transformation and recurrent respiratory infections, most suggest surgical resection at the time of diagnosis for the definitive treatment of symptomatic CPAM cases. In the pediatric population, surgical resection of all cystic lung lesions is generally recommended to prevent complications that may lead to more complex operation later on and also to pick up occult malignancies that are not identified preoperatively [33, 34]. For any age, the type and extent of surgical resection remain a debate. Traditionally lobectomy has been preferred because of the fear of incomplete removal of the pulmonary malformation [35] and complications like air leak associated with lung sparing surgeries [34]. Fascetti-Leon et al. in their retrospective review of 81 patients found lung sparing resection to be safe and effective with no increased risk of residual disease and recurrence if accurately planned in selected patients [36]. Bagrodia et al. came up with similar conclusion, while they suggested thoracotomy and possible lobectomy may still be necessary in cases with limited pulmonary reserve and larger malformations [35]. The resected specimen should always be carefully examined to look for occult malignancy [33]. Patients with bilateral CPAM with extensive lung involvement are mostly managed with conservative treatments, as surgery is risky and difficult [15]. Diagnosis in these cases can be confirmed by lung biopsy.

The treatment of asymptomatic CPAM is not well defined as the true incidence of complications in asymptomatic CPAM is unknown. Some authors argue against prophylactic surgeries stating that the risk of malignancy is overemphasized [2, 32]. They suggest close observation if the patient is agreeable after understanding the possible complications [32]. Also, prophylactic resection of CPAM lesions might not always be fully protective. Papagiannopoulos et al. state that prophylactic resection of lesions in CPAM patients does not protect them from later development of pleuropulmonary blastoma [34]. Even after resection of the lesion, it is recommended to closely observe patients for malignancy. Balkanli et al. described a case of bronchioloalveolar carcinoma in 19-year-old patient after undergoing resection of CPAM in infancy [32]. EGFR tyrosine kinase inhibitors can be beneficial in treating adenocarcinoma arising from type 1 CPAM with EGFR-mutation [16].

Survival rate at 6 months of age seems to have improved [14] probably because of the increased number of identified CPAM from the more common use of prenatal ultrasound. Prognoses among adult CPAM cases vary. Enuh et al. reported CPAM with aspergillosis in a 59-year-old male who died secondary to massive hemoptysis and development of disseminated intravascular coagulation during lobectomy [14]. Morelli et al. described CPAM in a 38-year-old male with persistent cough and hemoptysis who did well after lobectomy [20]. Because of the higher percentage of asymptomatic cases of CPAM and various degrees of lung involvement, it might be difficult to determine the prognosis in adults.

## 4. Conclusion

In otherwise healthy individuals presenting with recurrent pneumonias, causes for repeated infections need to be sought. If the same location is involved repeatedly, then any anatomic abnormality in the area needs to be considered. Careful review of history and images can reveal congenital lesions like CPAM. Though CPAM is extremely rare in adult patients, it should still be considered in the differential diagnosis of cystic lung disease. The prevalence of CPAM in adults might be higher than currently reported. CT scans are the initial diagnostic choices. Surgical resection prevents further episodes of infections and malignant transformation. Because of a small but definite risk of malignancy, it is also recommended to closely observe the individuals with CPAM for malignancy even after resection of the lesion.

## Figures and Tables

**Table 1:** 

	Type 0	Type 1	Type 2	Type 3	Type 4
Also called	Acinar dysplasia		Intermediate	Solid	

Frequency	1–3%	50–65%,	20–25%,	8%	10%

Relative frequency	Fifth	Most common	Second most common	Fourth	Third

Presumed site of development	Tracheobronchial	Bronchial or bronchiolar	Bronchiolar	Bronchiolar/alveolar	Distal acinar

Clinical presentation as adult	No reports	If smaller, may present later in life with recurrent infections (36 reported cases) [2, 15–19]	10 previously reported cases [2, 15]	No reports	One case [18]

Cyst size	0.5 cm	2 to 10 cm	<2–2.5 cm	<0.2 cm	Varying, up to 7 cm

Cyst lining	Ciliated pseudostratified	Cuboidal to pseudostratified columnar	Cuboidal to columnar, ciliated, may resemble ectatic bronchiole-like structures	Ciliated cuboidal, resembling fetal lung in canalicular stage	Types 1 and 2 alveolar, resembling bullous emphysema

Cyst wall	Connective tissue and vasculature	Broad fibromuscular connective tissue	Small amount of fibrovascular connective tissue	Usually solid	Thin, uniform, central loose vascular tissue

Other histologic findings	Bronchial-like structures, cartilaginous airways, smooth muscle	Cartilage islands, one-third showing mucous cells, sometimes in clusters	Entrapped bronchovascular bundles near edge of lesion; occasionally mature skeletal muscle	Solid, curved channels	Large cysts usually in peripheral lung

Risk of malignancy	Not identified	Bronchioloalveolar Carcinoma	Not identified	Not identified	Must rule out pleuropulmonary blastoma

Adapted from [2, 7, 10, 11].
